# Non-communicable diseases among low income adults in rural coastal communities in Eastern Sabah, Malaysia

**DOI:** 10.1186/s12889-019-6854-6

**Published:** 2019-06-13

**Authors:** Hazriani Harris, Yasmin B. H. Ooi, Jau-Shya Lee, Patricia Matanjun

**Affiliations:** Faculty of Food Science and Nutrition, Universiti Malaysia Sabah, Jalan UMS, 88400 Kota Kinabalu, Sabah Malaysia

**Keywords:** Non-communicable diseases, Rural, Coastal

## Abstract

**Background:**

Rural coastal communities in Sabah are still overly represented in the hardcore poor economic status. The aim of this study was to determine the prevalence of hypertension, diabetes mellitus and hypercholesterolemia among adults, in relation to economic status.

**Methods:**

A cross-sectional study using stratified random sampling was conducted in seven coastal villages in Semporna, Sabah: Kabogan Laut, Salimbangun, Pekalangan, Pokas, Tampi-Tampi Timbayan, Sum Sum and Selinggit. Socio-demographic data were obtained via interviewer administered questionnaires in Sabah Malay creole. Anthropometric measurements, blood pressure, fasting blood glucose and blood lipids were obtained.

**Results:**

A total of 330 adults (133 males, 197 females) completed the study. Mean age was 43.7 ± 15.8 years. Most participants (87%) were living below the Poverty Line Income. Median per capita household income was RM83.33/month (≈ USD20/month). The number of newly diagnosed cases of hypercholesterolemia was 40.6%, diabetes mellitus was 5.8%, and hypertension was 24.5%. Adults from the hardcore poor economic status (household income ≤RM760/month (≈USD183/month) were the most represented in those who did not have a blood pressure, blood sugar and blood lipids check in the 12 months preceding the study (Χ^2^, p < 0.01). Adults from hardcore poor economic status were also the most represented in undiagnosed hypertension and uncontrolled blood pressure among those diagnosed (Χ^2^, p = 0.013). Among diabetics from the hardcore poor group, the undiagnosed fasting blood glucose was 11.2 ± 4.5 compared to 5.1 ± 0.6 mmol/L for diagnosed diabetics (p < 0.001). Among hypercholesterolemics from the hardcore poor group, total cholesterol and LDL cholesterol values were significantly higher in the undiagnosed group compared to the diagnosed group (p < 0.001).

**Conclusion:**

Many people in this rural coastal community were unaware that they had high cholesterol level (40.6%) and elevated blood pressure (24.5%). Routine health check is not common among low income adults in rural coastal communities in Semporna. The findings suggest public health initiatives should emphasize access to and the necessity of routine health checks for those aged 40 years.

## Background

The current body of research into rural health in Malaysia showed 41.3% of indigenous adults in Sabah [1], 33.6% of adults in rural Kedah [2], and 29.8% of adults in rural Penang [3] had hypertension. The nationally representative National Health and Morbidity Survey 2015 showed overall hypertension among adults aged 18 years and older was 33.5% (95%CI: 31.6–35.4) in rural areas compared to 29.3% in urban areas (95%CI: 28.2–30.4). At the national level, prevalence was higher in males (30.7, 95%CI: 29.5–32.2) compared to females (29.7% (95%CI: 28.5–30.9) [4]. The REDISCOVER study investigated hypertension in urban and rural Malaysian communities from 2007 to 2011. It also reported that rural communities had higher prevalence of hypertension (51.2, 95%CI: 49.8–52.4) compared to urban communities (44.9, 95%CI: 43.6–46.2) [5]. In Sabah, the prevalence was reported as 26.8% (95% CI: 23.8–30.0), but the prevalence of undiagnosed hypertension was 13.9% (95% CI: 11.8–16.3) [4]. Nationally representative studies reported that the prevalence of hypertension in Sabah had been 29.1% (95% CI: 26.3–32.0) from NHMS 2011 and 29.9% (95%CI: 28.2–31.6) from NHMS 2006 [6]. For the same reporting period, the country prevalence were 32.7% (95% CI: 31.6–33.7) from NHMS 2011 [7] and 32.2% (95% CI: 31.6–32.8) from NHMS 2006 [8]. The trend was that prevalence of hypertension in the state of Sabah decreased from 2006 to 2015 and had been consistently lower than the country prevalence. At the national level, the prevalence of overall and undiagnosed hypertension were higher in lower income groups [4].

The NHMS 2015 reported that the prevalence of hypercholesterolemia was 47.7% in both rural and urban areas in the country. In Sabah, the prevalence was 40.9% (95%CI: 36.9–45.0), and the prevalence of undiagnosed hypercholesterolemia in the state was 32.6% (95%CI: 28.7–36.8) [4]. This was an increase from 31.1% (95%CI: 27.9–34.5) in Sabah from the NHMS 2011. The country wide prevalence from the NHMS 2011 was 32.7% (95%CI: 31.6–33.7) [7]. There was no available data from the NHMS 2006 and NHMS 1996 for the prevalence of hypercholesterolemia in Sabah [6]. Sabah had lower prevalence of hypercholesterolemia since nationally representative data were collected. Between NHMS 2011 and NHMS 2015, there was an increase in prevalence of hypercholesterolemia in Sabah. At the national level, the prevalence of overall and undiagnosed hypercholesterolemia were about the same in all income groups. In relation to gender, the prevalence were found to be significantly higher among females (52.2, 95% CI: 50.7–53.7) compared to males (43.5, 95% CI: 42.0–45.1) [4]. There was an increasing trend for all NCD risk factors. NHMS 2011 showed that at least 63% of adult Malaysians aged ≥18 years had at least one NCD risk factor, which were either overweight/obesity, high blood pressure, high blood sugar and high cholesterol [4].

The NHMS 2015 also reported that the prevalence of diabetes mellitus type II in rural Malaysia was 16.7% (95%CI: 15.4–18.1) compared to 17.7% (95%CI: 16.7–18.8) in urban areas. In Sabah, the prevalence was 14.2% (95%CI: 12.2–16.4), and the prevalence of undiagnosed diabetes in the state was 8.3% (95%CI: 6.7–10.3) [4]. Over the period of available nationally representative data, the prevalence of diabetes mellitus in Malaysia had been increasing: 15.2% (95% CI: 14.3–16.1) in NHMS 2011 [7], 14.9% for adults aged ≥30 years in NHMS 2006 and 8.3% adults aged ≥30 years in NHMS 1996 [9]. For the corresponding period, the prevalence in Sabah from available data were 9% (95% CI: 7.2–11.3) in NHMS 2011 and 4.9% in NHMS 2006 [6]. Sabah had lower prevalence of diabetes mellitus compared to the national prevalence, but there was an increase in prevalence from 2006 to 2015. At the national level, the prevalence of overall and undiagnosed diabetes were higher in lower income groups [4]. The most recent NHMS 2015 showed that prevalence of diabetes was higher in females (18.3, 95%CI 17.2–19.4) compared to males (16.7, 95%CI: 15.7–17.8): [4].

Nationally representative data showed that the prevalence of non-communicable diseases (NCDs) have continued to rise for the last two decades in Malaysia. In response to that, the Ministry of Health implemented the National Strategic Plan for Non-Communicable Diseases (NSP-NCD) 2010–2014 and the NCD Prevention 1 Malaysia (NCDP-1 M) programmes. Through the NCDP-1 M, the government health services took the approach of engaging the community as a partner in prevention and promotion, such as NCD risk factor screening and intervention in the community, workplace and schools [10, 11]. The NCDP-1 M was project based and focused on NCD risk factors. Each state determined its own NCDP-1 M project decisions. The limitations were that the quality of the projects and implementations were inconsistent, inadequate human resources and increased burden to existing services [10]. In comparison, China as a middle income country which also has an increasing NCD prevalence and burden like Malaysia, focused on comprehensive interventions in high-risk population. Like Malaysia’s NCDP-1 M programme, China encouraged its local governments to develop their own strategies and measures. In contrast, Japan as a high income country has a stable NCD prevalence. Its main strategy for NCDs control is primary prevention, with a universal NCDs prevention programme for all adults aged 40–74 years [12]. Japanese on low income had better access to the universal health care system in Japan compared to their Chinese counterparts in China. The social health insurance system in China did not prioritise outpatient costs, which would have been the most needed service for NCD patients [12]. It is important for governments to have adequate budget for prevention and promotion activities via effective channels.

Data for the state of Sabah might not give a representative idea on the health status of a rural coastal community such as Semporna. Semporna’s most dominant ethnic group is the Bajau, which constitutes 57% of the population in that district [13]. They mostly speak the Sabah Malay creole and Bajau. The Bajaus consist of individuals who hold Malaysian citizenship and those who do not. The former are also known as the Bajau Tempatan (‘emplaced Bajau’) and the latter are known as the Bajau Laut. The two groups are also labelled within Sabah as simply the ‘Bajau’. The Malaysian census reported that Semporna had a population of 133,164 comprising of Malaysian citizens and non-citizens [13]. Individuals who hold Malaysian citizenship have often oriented themselves to terrestrial livelihoods whilst maintaining some orientation to the marine based livelihoods, for example seaweed cultivation and fishing [14]. These groups are also more sedenterised than the Bajau Lauts who continue to ply the seas in their houseboats.

Many parts of Sabah, East Malaysia are considered rural and low income in terms of development. Populations here face significant health and nutritional challenges often observed in low-income situations. Rural health is often approached as challenges and strategies within the context of inaccessible interior regions [15] and less often within the context of coastal regions far from urban areas. However, Sabah being about half the size of Peninsular Malaysia, has challenges in health access for populations in interior regions as well as its vast coastal regions and inhabited islands. Verbal communication among health professionals indicated that non-communicable diseases in coastal regions in Sabah are largely undiagnosed, but there is no published evidence. We sought to fill this gap by conducting a cross-sectional study in Semporna, Sabah. Semporna is on the southeast of Sabah, facing the Sulawesi Sea. A similar gap in absence of NCD prevalence data for the island and coastal areas in China also presented challenges in confirming the authenticity of their published statistics [16]. Elucidation of NCD prevalence and observation of socio-economic situations in diagnosed and undiagnosed individuals will support more effective public health strategies.

## Methods

### Study design and sampling

This cross-sectional study was carried out in seven coastal villages on the Semporna mainland. All heads of coastal villages on the Semporna mainland that were deemed safe to visit by a local informant were visited and briefed about the study. Many areas were not accessible during the field work period because of recent armed incursions by foreigners and the resultant military operations by the Malaysian Armed Forces [17]. The local social dynamics were such that potential researchers and respondents should obtain the approval of village heads before commencement of studies. The village heads who agreed to participate provided information about number of households in their village. Based on the information gathered, invitation fliers were distributed by hand to each household (n = 355). The villages that agreed to participate were Kabogan Laut, Salimbangun, Pekalangan, Pokas, Tampi-Tampi Timbayan, Sum Sum and Selinggit. The inclusion criteria were that potential respondents aged ≥19 years were able to communicate in Malay or Sabah Malay creole, without mental illness and physical disability, were not pregnant or lactating.

Sample size was calculated as n = Z^2^P(1-P)/d^2^, where p = 0.291 based on the prevalence of hypertension of 29.1% for adults aged ≥18 years in Sabah [7]. Published national data only showed prevalence by states in the federation of Malaysia. Discussions with the district nutritionist indicated that hypertension and diabetes were prevalent among adults in Semporna. As the prevalence for diabetes in Sabah was 9.0% [7], the sample size was calculated using the prevalence of hypertension to generate a larger sample size. At 95% level of confidence and 5% precision, therefore n = 1.96^2^ X 0.291 (1–0.291)/0.05^2^, a sample size of 317 adults were calculated. Stratified random sampling method was employed to recruit the respondents. There were an estimate of 1420 adults aged ≥19 years in the seven villages. The population was stratified according to age group: adults aged 19–59 years and elderly aged ≥60 years to ensure we had respondents from the older adults. Respondents were recruited randomly from each stratum. Fliers were distributed throughout daylight hours everyday until all houses in all participating villages were visited. When nobody was at home, the household was visited again so that fliers could be handed in person and study information could be provided face to face and invitation to participate could be conveyed. Randomness was achieved as every household had equal chances of being visited at any daylight hour, and any potential respondent could be home at that the time of the visit. A maximum of one adult and one elderly person from each household within the inclusion criteria could freely participate as respondents. A total of 330 individuals (284 (86.1%) individuals aged 19–59 years; 46 (13.9%) individuals aged ≥60 years) completed the study procedures. Ethics approval was obtained from the Medical Research Ethics Committee, Faculty of Medicine and Health Sciences, Universiti Malaysia Sabah. The approval code is JKEtika 3/14(3). Preliminary groundwork to seek permissions at the study location was conducted from December 2014 to January 2015. All respondents were interviewed and biological samples were collected from February to May 2015.

### Study procedures

Written informed consent was obtained from each respondent before they began their participation. Socio-demography and medical history data were obtained using interviewer-administered questionnaires. One researcher interviewed all respondents in their respective households. The socio-demographic data recorded were gender, age, ethnic group, religious affiliation, educational level, marital status, occupation, household income, household size and cigarette/tobacco smoking status. Medical history data were obtained using an adapted questionnaire [7]. Respondents were asked regarding family history and whether they had attended a health check in the past 12 months or diagnosed with hypertension, hypercholesterolemia and/or diabetes mellitus. Respondents were then given weekend appointments from 8 to 11 a.m. at the village hall for anthropometric and blood pressure measurements and biological sampling. Body mass index (BMI), body fat, waist circumference, blood pressure, and fasting venous blood sample were obtained during this appointment. Venous blood samples were collected by a qualified health professional to determine fasting blood glucose, HDL-cholesterol, LDL-cholesterol, total cholesterol and triglycerides. Respondents were reminded to fast overnight for at least 8 h prior to blood sample collection. Plain water consumption was not restricted throughout that period. Respondents were asked to confirm their fasting status before a blood sample was taken. If they had not fasted or were feeling unwell, they were given another appointment for the following weekend. All blood samples were analysed in the accredited BP Diagnostic Centre, Kota Kinabalu, Sabah, Malaysia using ARCHITECT c800 Clinical Chemistry Analyser. All respondents were informed in writing of their anthropometric, blood pressure and blood sampling results. When diagnosed as hypertensive, hypercholesterolemic and/or diabetic, they were informed to seek a physician’s attention with their results.

### Socio-demography

Respondents were asked to state their ethnic group and religious affiliations. Educational levels were classified into five categories as ‘no formal education’, ‘primary’, ‘lower secondary’, ‘upper secondary’, ‘foundation, matriculation, high school certificate, diploma or equivalent’, and ‘undergraduate degree’. Marital status was classified into four categories as ‘single’, ‘married’, ‘divorced or separated’, and ‘widow/widower’. Occupation was classified into four categories as ‘not working or unemployed’, ‘home maker’, ‘self-employed’ and ‘public or private employee’. Cigarette smoking status was classified into three categories as ‘do not smoke’, ‘have smoked in the past’ and ‘smoking’. The former two categories were merged as ‘non- or ex-smoker’ in data analysis.

Household income was classified into three categories as ‘hardcore poor, with a household income of ≤RM760/month’, ‘poor, with a household income of RM761 – 1,180/month’ and ‘above rural Poverty Line Income’. The hardcore poor cut-off of ≤RM760/month and Poverty Line Income of RM1,180/month for households in rural Sabah were obtained from the Economic Planning Unit, a Malaysian government agency, and based on monitoring data from January to December 2014 [18]. The Poverty Line Income (PLI) was calculated based on the minimum requirements for basic food and non-food items of every individual in a household. The basic food items were based on the Recommended Nutrient Intake and physical activity level. The non-food items were based on clothing, shoes, accommodation, fuel, utilities, basic household furniture and appliances, transportation and communication as identified by the Household Expenditure Surveys. Hardcore poverty was defined as income that is less than the cost of the basic food items [18].

### Anthropometric measurements

The anthropometric measurements were conducted based on standard procedures. Height was measured to the nearest 0.1 cm using SECA Portable Stadiometer 213. Weight was measured to the nearest 0.1 kg and body fat was given as percentage of body fat using OMRON Karada Scan Body Composition Monitor HBF-375. Respondents were measured in light clothing without shoes. BMI was calculated as kg/m^2^, and the WHO (2004) cut-offs were used: underweight (< 18.5 kg/m^2^), normal (18.5–22.9), overweight/pre-obese (23.0–27.4), obese I (27.5–34.9), obese II (35.0–39.9) and obese III (≥40.0) [19].

Percent body fat cut-offs used were: ‘low body fat’ [(20–39 years: < 13% for men and < 25% for women), (40–59 years: < 13% for men and < 25% for women) and (60–79 years: < 14% for men and < 25% for women)], ‘normal body fat’ [(20–39 years: 13–22% for men and 25–34% for women), (40–59 years: 13–23% for men and 25–34% for women) and (60–79 years: 14–23% for men and 25–35% for women)], ‘moderate body fat’ [(20–39 years: 23–27% for men and 35–39% for women), (40–59 years: 24–28% for men and 35–40% for women) and (60–79 years: 24–28% for men and 36–40% for women)] and ‘high body fat [(20–39 years: ≥28% for men and ≥ 40% for women), (40–59 years: ≥29% for men and ≥ 41% for women) and (60–79 years: ≥29% for men and ≥ 41% for women)] [20].

Waist circumference was measured to the nearest 0.1 cm using SECA Measuring Tape 201 to determine abdominal obesity. The cut-offs were: ‘normal waist circumference’ (< 90 cm for men and < 80 cm for women) and ‘positive abdominal obesity’ (≥90 cm for men and ≥ 80 cm for women) [21].

### Blood pressure measurements

Blood pressure was measured using OMRON Automatic Blood Pressure Monitor SEM-1. Respondents were measured in a rested and seated condition at their village hall. At least two measurements were performed at five minutes apart on respondents’ right arm rested on a table at heart level. If the two systolic and diastolic readings did not differ by more than 5 mmHg, the average of these values was used as the blood pressure value for that individual. If the readings from the initial two measurements differed more than 5 mmHg, subsequent measurements were taken 5–10 min later until two values within the acceptable difference were obtained. The Malaysian Ministry of Health’s Clinical Practice Guidelines for Hypertension cut-offs for blood pressure were used: ‘optimal’ (< 120 mmHg for systolic and < 80 mmHg for diastolic), ‘normal’ (< 130 mmHg for systolic and < 85 mmHg for diastolic), ‘high normal’ (130–139 mmHg for systolic and/or 85–89 mmHg for diastolic), ‘hypertension I’ (140–159 mmHg for systolic and/or 90–99 mmHg for diastolic), ‘hypertension II’ (160–179 mmHg for systolic and/or 100–109 mmHg for diastolic) and ‘hypertension III’ (≥180 mmHg for systolic and/or ≥ 110 mmHg for diastolic) [22].

### Blood glucose and lipids cut-offs

The Malaysian Ministry of Health’s Clinical Practice Guidelines on type 2 diabetes mellitus cut-offs for fasting blood glucose were used: ‘normal blood glucose’ (< 7.0 mmol/L) and ‘positive diabetes mellitus’ (≥7.0) [23]. The blood lipids cut-offs from the National Cholesterol Education Programme were used. Total cholesterol cut-offs were: ‘normal’ (< 5.2 mmol/L), ‘borderline high’ (5.2–6.1) and ‘high’ (≥6.2). Triglyceride cut-offs were: ‘normal’ < 1.7 mmol/L, ‘borderline high’ (1.7–2.2 mmol/L), ‘high’ (2.3–5.6 mmol/L) and ‘very high’ (≥5.7 mmol/L). LDL cholesterol cut-offs were: ‘normal’ (< 2.6 mmol/L), ‘above normal’ (2.6–3.3 mmol/L), ‘borderline high’ (3.4–4.1 mmol/L), ‘high’ (4.2–4.8 mmol/L) and ‘very high’ (≥4.9 mmol/L). HDL cholesterol cut-offs were: ‘low’ (< 1.0 mmol/L), ‘normal’ (1.0–1.5 mmol/L) and ‘high’ (≥1.6 mmol/L) [24].

### Statistical analyses

All data were analysed using IBM SPSS Statistics 24. Kolmogorov-Smirnov test was used to assess for the Normal distribution; P > 0.05 was considered to be Normally distributed. The results were presented as frequencies (N) and percentages (%) for categorical variables and as means and standard deviations (S.D.) for continuous variables. Chi-square test was used to determine associations between categorical variables. Binary logistic regression was attempted with each disease as the dependent variable; and gender, age group, education level, household income level, occupation categories, waist circumference and BMI as independent variables in the full model. Unpaired t-test and Mann-Whitney test were used to determine differences between two groups for continuous variable. One-way ANOVA with Tukey’s b post hoc test and Kruskal-Wallis test were used to determine differences between three or more groups for continuous variables. P < 0.05 was considered to be statistically significant.

## Results

### Socio-demography

A total of 330 adults (133 males, 197 females) participated in this study out of 355 individuals who were approached during the door-to-door recruitment. There were mostly one respondent from each household. The response rate was about 93%. Mean age was 43.7 ± 15.8 years. Most of the respondents were individuals aged 30–59 years (64.8%) and described themselves as belonging to the Bajau ethnic group (98.2%). All respondents described themselves as Muslims. Most respondents were educated to secondary school level (47.9%). The men were mainly self-employed (n = 83, 62.4%). Most self-employed men were fishermen (n = 32, 38.6% of those self-employed), private bus drivers (n = 15, 18.15%) and farmers (n = 11, 13.3%). The women were mainly housewives (n = 144, 73.1%). Household income were mostly below Poverty Line Income for households in rural Sabah of RM1, 180/month [18]; 14.2% were poor (household income RM761–1180/month) and 72.4% were hardcore poor (household income ≤RM760/month). Most households in Semporna had more than 6 individuals per household (60.9%). Median per capita household income was RM83.33/month. Only 11.5% of respondents smoked cigarettes. More details are reported in Table 1.

**Table 1:** Socio-demographic characteristics of respondents

Characteristics	Sex, N (column %)				All (*n* = 330)	
	Men (*n* = 133)		Women (*n* = 197)			
	N	(%)	N	(%)	N	(%)
Age group (years)	43.0 ± 16.4^a^		44.1 ± 15.4^a^		43.7 ± 15.8^a^	
19–29	32	(24.1)	38	(19.3)	70	(21.3)
30–59	80	(60.2)	134	(68.0)	214	(64.8)
≥ 60	21	(15.8)	25	(12.7)	46	(13.9)
Ethnic group						
Bajau	131	(98.5)	193	(98.0)	324	(98.2)
Bugis	0	(0.0)	2	(1.0)	2	(0.6)
Jawa	1	(0.8)	0	(0.0)	1	(0.3)
Suluk	1	(0.8)	1	(0.5)	2	(0.6)
Sungai	0	(0.0)	1	(0.5)	1	(0.3)
Religion						
Islam	133	(100.0)	197	(100.0)	330	(100.0)
Educational level						
No formal education	20	(15.0)	63	(32.0)	83	(25.2)
Primary school	26	(19.5)	39	(19.8)	65	(19.7)
Secondary school	75	(56.4)	83	(42.1)	158	(47.9)
High school certificate / Diploma	7	(5.3)	9	(4.6)	16	(4.8)
Bachelor’s degree	5	(3.8)	3	(1.5)	8	(2.4)
Marital status						
Single	39	(29.3)	38	(19.3)	77	(23.3)
Married	88	(66.2)	128	(65.0)	216	(65.5)
Divorced/Separated	0	(0.0)	2	(1.0)	2	(0.6)
Widow/Widower	6	(4.5)	29	(14.7)	35	(10.6)
Occupation						
Unemployed	32	(24.1)	31	(15.7)	63	(19.1)
Housewife	0	(0.0)	144	(73.1)	144	(43.6)
Self-employed	83	(62.4)	19	(9.6)	102	(30.9)
Employed	18	(13.5)	3	(1.5)	21	(6.4)
Household income (RM/month)	750.0 ± 713.0^a^		618.8 ± 443.6^a^		671.7 ± 569.9^a^	
Hardcore poor	97	(72.9)	142	(72.1)	239	(72.4)
Poor	15	(11.3)	32	(16.2)	47	(14.2)
Above PLI	21	(15.8)	23	(11.7)	44	(13.3)
Household size (Number of individuals)	6.4 ± 3.1^a^		6.8 ± 3.2^a^		6.7 ± 3.2^a^	
1–5	59	(44.4)	70	(35.5)	129	(39.1)
≥ 6	74	(55.6)	127	(64.5)	201	(60.9)
Smoking status						
Smoker	31	(23.3)	7	(3.6)	38	(11.5)
Non/Ex-smoker	102	(76.7)	190	(96.4)	292	(88.5)

^a^ Values are in mean ± S.D.

### Non-communicable diseases

A total of 108 (32.7%) respondents were identified as hypertensive; 81 respondents (24.5%) had never been diagnosed by a health professional. Of those who had been previously diagnosed (n = 27), 11 (40.7%) had well controlled blood pressure, and 16 (59.3%) had uncontrolled blood pressure readings. There were 225 individuals (68.2%) who had not had their blood pressure checked in the preceding 12 months. Of these 225 individuals, 64 (28.4%) were found to have undiagnosed elevated blood pressure. Of those with undiagnosed elevated blood pressure, 79.0% were those who did not have a blood pressure check (*p* < 0.001).

The number of hypercholesterolemic respondents was 140 (42.4%); 134 respondents (40.6%) had never been diagnosed by a health professional. Of those who had been previously diagnosed (n = 6), only 1 (16.7%) respondent had well controlled total cholesterol levels, and 5 (83.3%) had poorly controlled total cholesterol levels. There were 303 individuals (91.8%) who had not had their blood lipids checked in the preceding 12 months. Of these 303 individuals, 129 (42.6%) were found to have undiagnosed elevated total cholesterol. Among those who were undiagnosed, 96.3% were those who did not have a blood lipids check (*p* < 0.001).

A total of 24 respondents (7.3%) were identified as diabetic; 19 respondents (5.8%) had never been diagnosed by a health professional. Of those who had been previously diagnosed (n = 5), 3 (60%) respondents had well controlled fasting blood glucose and 2 had poorly controlled levels (40%). There were 287 individuals (87.0%) who had not had their blood glucose checked in the preceding 12 months. Of these 287 individuals, 17 (5.9%) were found to have undiagnosed elevated fasting blood glucose. Among those who were undiagnosed, 89.5% were those who did not have a blood glucose check (p < 0.001).

As expected, older individuals were more represented among those with hypertension, hypercholesterolemia and diabetes mellitus (p = 0.001). For hypertension, the OR for respondents aged 30 – 59y was 0.34 (95% CI: 0.14–0.79, p = 0.012) compared to respondents aged ≥60y. The ≥60y age group had the highest number of individuals with hypertension. For diabetes mellitus, the OR for respondents aged 30 – 59y was 0.21 (95% CI: 0.06–0.75, p = 0.016) compared to respondents aged ≥60y. The 30 – 59y age group had the highest number of individuals with diabetes mellitus and hypercholesterolemia. More details are shown in Tables 2 and 3.

**Table 2:** Prevalence of hypertension (HPN), hypercholesterolemia (HPC) and diabetes mellitus (DM) by socio-demography of respondents

Risk factors	Hypertension, N (row %)				Chi-square/*p*-value	Hypercholesterolemia, N (row %)				Chi-square/*p*-value	Diabetes Mellitus, N (row %)				Chi-square/*p*-value
	HPN (*n* = 108)		Non-HPN (*n* = 222)			HPC (*n* = 140)		Non-HPC (*n* = 190)			DM (*n* = 24)		Non-DM (*n* = 306)		
	N	(%)	N	(%)		N	(%)	N	(%)		N	(%)	N	(%)	
Sex					0.133/					0.303/					0.523/
Men	42	(31.6)	91	(68.4)	0.715	54	(40.6)	79	(59.4)	0.582	8	(6.0)	125	(94.0)	0.524
Women	66	(33.5)	131	(66.5)		86	(43.7)	111	(56.3)		16	(8.1)	181	(91.9)	
Age group (years)					32.226/					21.754/					13.058/
19–29	9	(11.2)	71	(88.8)	< 0.001	16	(20.0)	64	(80.0)	< 0.001	0	(0.0)	80	(100.0)	0.001
30–59	75	(35.5)	136	(64.5)		105	(49.8)	106	(50.2)		17	(8.1)	194	(91.9)	
≥ 60	24	(61.5)	15	(38.5)		19	(48.7)	20	(51.3)		7	(17.9)	32	(82.1)	
Educational level					32.218/					34.319/					5.237/
No formal education	47	(56.6)	36	(43.4)	< 0.001	56	(67.5)	27	(32.5)	< 0.001	9	(10.8)	74	(89.2)	0.264
Primary school	21	(32.3)	44	(67.7)		26	(40.0)	39	(60.0)		5	(7.7)	60	(92.3)	
Secondary school	37	(23.4)	121	(76.6)		52	(32.9)	106	(67.1)		10	(6.3)	148	(93.7)	
High School Certificate/Diploma	2	(12.5)	14	(87.5)		2	(12.5)	14	(87.5)		0	(0.0)	16	(100.0)	
Bachelor’s degree	1	(12.5)	7	(87.5)		4	(50.0)	4	(50.0)		0	(0.0)	8	(100.0)	
Marital status					19.410/					21.696/					8.725/
Single	12	(15.6)	65	(84.4)	< 0.001	18	(23.4)	59	(76.6)	< 0.001	1	(1.3)	76	(98.7)	0.033
Married	76	(35.2)	140	(64.8)		100	(46.3)	116	(53.7)		18	(8.3)	198	(91.7)	
Divorced/Separated	1	(50.0)	1	(50.0)		0	(0.0)	2	(100.0)		0	(0.0)	2	(100.0)	
Widow/Widower	19	(54.3)	16	(45.7)		22	(62.9)	13	(37.1)		5	(14.3)	30	(85.7)	
Occupation					11.521/					11.494/					3.375/
Unemployed	11	(17.5)	52	(82.5)	0.009	15	(23.8)	48	(76.2)	0.009	2	(3.2)	61	(96.8)	0.337
Housewife	56	(38.9)	88	(61.1)		70	(48.6)	74	(51.4)		12	(8.3)	132	(91.7)	
Self-employed	31	(30.4)	71	(69.6)		46	(45.1)	56	(54.9)		6	(5.9)	96	(94.1)	
Employed	10	(47.6)	11	(52.4)		9	(42.9)	12	(57.1)		1	(4.8)	20	(95.2)	
Household income (RM/month)					0.450/					0.837/					0.095/
Hardcore poor	78	(32.6)	161	(67.4)	0.799	101	(42.3)	138	(57.7)	0.658	18	(7.5)	221	(92.5)	0.954
Poor	14	(29.8)	33	(70.2)		18	(38.3)	29	(61.7)		3	(6.4)	44	(93.6)	
Above PLI	16	(36.4)	28	(63.6)		21	(47.7)	23	(52.3)		3	(12.5)	41	(93.2)	
Household size (People)					0.035/					0.724/					0.360/
1–5	43	(33.3)	86	(66.7)	0.851	51	(39.5)	78	(60.5)	0.395	8	(6.2)	121	(93.8)	0.666
≥ 6	65	(32.3)	136	(67.7)		89	(44.3)	112	(55.7)		16	(8.0)	185	(92.0)	
Smoking status					0.043/ 0.836					1.832/ 0.176					1.372/ 0.334
Smoker	13	(34.2)	25	(65.8)		20	(52.9)	18	(47.4)		1	(2.6)	37	(97.4)	
Non/Ex-smoker	95	(32.5)	197	(67.5)		120	(41.1)	172	(58.9)		23	(7.9)	269	(92.1)

**Table 3:** Odd ratios for hypertension, hypercholesterolemia and diabetes mellitus

	Hypertension (yes vs. no), Nagelkerke R^2^ = 0.351), overall predictive accuracy = 75.5%			Hypercholesterolemia (yes vs. no), Nagelkerke R^2^ = 0.253), overall predictive accuracy = 68.2%			Diabetes mellitus^a^ (yes vs. no), Nagelkerke R^2^ = 0.270, overall predictive accuracy = 93.0%		
	OR	(95% CI)	P value	OR	(95% CI)	P value	OR	(95% CI)	P value
Education level with undergraduate degree as the reference									
No formal education	40.1	(2.77–581.52)	0.007	2.66	(0.33–21.62)	0.361			
Primary	15.4	(1.09–217.12)	0.043	0.74	(0.10–5.64)	0.769			
Lower secondary	14.5	(1.06–197.14)	0.045	0.74	(0.10–5.40)	0.758			
Upper secondary	14.1	(1.02–196.13)	0.048	0.44	(0.06–3.30)	0.427			
High school certificate /diploma	13.7	(0.57–326.01)	0.106	0.24	(0.02–3.00)	0.262			
Household income with above PLI as the reference									
Hardcore poor	0.48	(0.18–1.27)	0.139	0.34	(0.14–0.85)	0.020	0.56	(0.09–3.36)	0.525
Poor	0.53	(0.17–1.60)	0.256	0.48	(0.17–1.34)	0.161	0.65	(0.08–5.20)	0.688
Occupation with public / private employee as the reference									
Unemployed	0.27	(0.05–1.47)	0.129	2.04	(0.42–9.79)	0.375	3.83	(0.15–99.29)	0.418
Home maker	0.29	(0.05–1.79)	0.182	2.23	(0.44–11.45)	0.336	3.43	(0.12–101.34)	0.476
Self employed	0.28	(0.06–1.31)	0.106	2.64	(0.62–11.22)	0.187	1.98	(0.12–33.22)	0.634
Age with ≥60y as the reference									
19 – 29y	0.37	(0.10–1.40)	0.143	1.70	(0.52–5.57)	0.384	0	0	0.997
30 – 59y	0.34	(0.14–0.79)	0.012	1.59	(0.71–3.58)	0.260	0.21	(0.06–0.75)	0.016
Gender with females as the reference									
Males	1.96	(0.62–6.15)	0.252	2.44	(0.92–6.48)	0.074	2.16	(0.26–17.67)	0.473
Waist circumference (cm)	1.09	(1.04–1.14)	< 0.001	1.04	(1.00–1.08)	0.085	1.07	(1.00–1.15)	0.044
BMI (kg/m^2^)	1.20	(0.77–1.86)	0.543	0.86	(0.58–1.29)	0.472	1.09	(0.41–2.90)	0.867

^a^ OR for education level for diabetes was not reported as there were no respondents who were diabetic with foundation / high school / diploma and undergraduate degree levels

Adults with lower educational levels were more represented among those with hypertension and hypercholesterolemia (*p* < 0.001) (Table 2). For hypertension, the OR for respondents with no formal education was 40.1 (95% CI: 2.77–581.52, p = 0.007) compared to respondents with an undergraduate degree level of education. The OR for hypertension decreased as education level increased (Table 3).

Adults form hardcore poor households were the most represented in those who did not have a blood pressure, blood sugar and blood lipids check in the 12 months preceding this present study (*p* < 0.01). More details are reported in Table 4. Individuals living in hardcore poor households who had not been previously diagnosed with NCDs also had significantly higher systolic blood pressure, total cholesterol, LDL-cholesterol and fasting blood glucose compared to individuals in similar economic situation who were without the NCD or had been diagnosed with it (*p* < 0.001). Fasting blood glucose was significantly higher in non-diabetic individuals living in hardcore poor households compared to non-diabetic individuals in poor and above PLI households (*p* = 0.006). More details are reported in Table 5. Respondents who were obese, had very high body fat percentage and positive abdominal obesity were significantly represented among those who had hypertension, hypercholesterolemia and diabetes mellitus (*p* < 0.001). More details are reported in Table 6.

**Table 4:** Access to health checks by economic status of households

	Economic status of household	Had a health check in the preceding 12 months		Chi square test, p value
		No, n (row%)	Yes, n (row %)	
Blood pressure check	Hardcore poor	178 (74.5)	61 (25.5)	< 0.001
	Poor	30 (63.8)	17 (36.2)	
	Above PLI	17 (38.6)	27 (61.4)	
Blood lipids check	Hardcore poor	226 (94.6%)	13 (5.4)	0.009
	Poor	42 (89.4)	5 (10.6)	
	Above PLI	35 (79.5)	9 (20.5)	
Blood glucose check	Hardcore poor	220 (92.1)	19 (7.9)	< 0.001
	Poor	36 (76.6)	11 (23.4)	
	Above PLI	31 (70.5)	13 (29.5)

**Table 5:** Blood pressure and blood chemistry by status of diagnosis of hypertension (HPN), hypercholesterolemia (HPC) and diabetes mellitus (DM) according to household income levels

		Hardcore poor			Poor			Above PLI			*p* value^A,B^
		N	Mean ± S.D.	*p* value^a,b^	N	Mean±S.D.	*p* value^a,b^	N	Mean±S.D.	*P* value^a,b^	
Systolic BP (mmHg)	No HPN	161	118.5±11.2^aA^	< 0.001	33	117.5±10.1^aA^	< 0.001	28	118.5±12.5^aA^	< 0.001	0.883
	Known HPN	13	140.8±19.9^bA^		4	133.5±19.0^bA^		10	159.6±30.4^bA^		0.113
	Unknown HPN	65	155.4±18.9^cA^		10	147.2±6.7^cA^		6	157.3±14.7^bA^		0.371
Diastolic BP (mmHg)	No HPN	161	71.8±8.1^aA^	< 0.001	33	72.9±7.8^aA^	0.010	28	71.3±8.8^aA^	< 0.001	0.686
	Known HPN	13	83.7±12.9^bA^		4	81.3±11.6^aA^		10	88.2±12.5^bA^		0.574
	Unknown HPN	65	86.6±9.0^bA^		10	81.9±9.9^aA^		6	90.2±11.4^bA^		0.195
Total cholesterol (mmol/L)	No HPC	138	4.3±0.6^aA^	< 0.001	29	4.3±0.5^A^	< 0.001	23	4.2±0.6^aA^	< 0.001	0.858
	Known HPC	2	4.8±0.9^a^		1	8.1		3	6.4±0.6^b^		0.068
	Unknown HPC	99	6.2±0.8^b^		17	5.9±0.5^A^		18	6.2±0.8^bA^		0.319
HDL-C (mmol/L)	No HPC	138	1.2±0.2^aA^	0.004	29	1.3±0.2^A^	0.070	23	1.3±0.3^aA^	0.774	0.390
	Known HPC	2	1.3±0.1^a^		1	1.4		3	1.3±0.2^a^		0.867
	Unknown HPC	99	1.3±0.3^aA^		17	1.5±0.3^A^		18	1.3±0.2^aA^		0.164
LDL-C (mmol/L)	No HPC	138	2.5±0.5^aA^	< 0.001	29	2.5±0.4^A^	< 0.001	22	2.5±0.6^aA^	< 0.001	0.228
	Known HPC	2	3.0±0.7^a^		1	5.6		3	4.2±0.6^b^		0.806
	Unknown HPC	98	4.1±0.7^bA^		17	3.8±0.5^A^		18	4.1±0.6^bA^		0.124
Trg (mmol/L)	No HPC	138	1.2±0.6^aA^	< 0.001	29	1.1±0.5^A^	0.025	23	1.1±1.0^aA^	0.045	0.817
	Known HPC	2	1.1±0.3^a^		1	2.5		3	2.1±0.4^a^		0.096
	Unknown HPC	99	1.8±1.0^aA^		17	1.4±0.6^A^		18	1.8±0.8^aA^		0.367
FBG (mmol/L)	No DM	221	5.0±0.7^aA^	< 0.001	44	4.8±0.7^A^	< 0.001	41	4.7±0.6^A^	< 0.001	0.006
	Known DM	3	5.1±0.6^a^		1	12.2		1	19.7		0.005
	Unknown DM	15	11.2±4.5^bA^		2	15.7±6.6^A^		2	10.8±4.6^A^		0.446

^a,b^Different superscripts within the same column denote significant difference between NCD status (*p* < 0.05) for each blood chemistry. The group sizes are unequal. Type I error levels might occur for the ANOVA

^A, B^ Different superscripts within the same row denote significant difference between household income levels (*p* < 0.05) for each blood chemistry. The group sizes are unequal. Type I error levels might occur for the ANOVA

**Table 6:** Prevalence of hypertension (HPN), hypercholesterolemia (HPC) and diabetes mellitus (DM) by nutritional status of respondents

Risk factors	Hypertension, N (row %)				Chi-square/ p-value	Hypercholesterolemia, N (row %)				Chi-square/ p-value	Diabetes Mellitus, N (row %)				Chi-square/ p-value
	HPN (*n* = 108)		Non-HPN (n = 222)			HPC (n = 140)		Non-HPC (n = 190)			DM (n = 24)		Non-DM (n = 306)		
	N	(%)	N	(%)		N	(%)	N	(%)		N	(%)	N	(%)	
Body Mass Index (BMI)					29.426/					29.120/					20.015/
Underweight (< 18.5 kg/m^2^)	2	(5.9)	32	(94.1)	< 0.001	6	(17.6)	28	(82.4)	< 0.001	0	(0.0)	34	(100.0)	< 0.001
Normal (18.5–22.9 kg/m^2^)	18	(19.8)	73	(80.2)		24	(26.4)	67	(73.6)		0	(0.0)	91	(100.0)	
Overweight (23–27.4 kg/m^2^)	39	(38.6)	62	(61.4)		52	(51.5)	49	(48.5)		8	(7.9)	93	(92.1)	
Obese (≥27.5 kg/m^2^)	49	(47.1)	55	(52.9)		58	(55.8)	46	(44.2)		16	(15.4)	88	(84.6)	
Body Fat (BF)					39.055/< 0.001					34.919/					21.404/< 0.001
Low BF	1	(2.6)	38	(97.4)		4	(10.3)	35	(89.7)	< 0.001	0	(0.0)	39	(100.0)	
Normal BF	33	(24.3)	103	(75.7)		48	(35.3)	88	(64.7)		3	(2.2)	133	(97.8)	
Moderate BF	49	(44.5)	61	(55.5)		59	(53.6)	51	(46.4)		13	(11.8)	97	(88.2)	
High BF	25	(56.8)	19	(43.2)		29	(65.9)	15	(34.1)		8	(18.2)	36	(81.8)	
Waist Circumference (WC)					22.936/< 0.001					25.991/					16.401/< 0.001
Positive abdominal obesity (Men: ≥90 cm; Women: ≥80 cm)	86	(42.6)	116	(57.4)		108	(53.5)	94	(46.5)	< 0.001	24	(11.9)	178	(88.1)	
Normal WC (Men: < 90 cm; Women: < 80 cm)	22	(17.2)	106	(82.8)		32	(25.0)	96	(75.0)		0	(0.0)	128	(100.0)	

## Discussion

Hypercholesterolemia (42.4%) was the most prevalent NCD, followed by hypertension (32.7%) and diabetes mellitus (7.3%) in Semporna, Sabah. These are comparable to the national prevalence in rural areas of 47.7% for hypercholesterolemia and 33.5% for hypertension [4]. The prevalence for diabetes mellitus in Semporna was half of the national prevalence in rural areas of 16.7% for diabetes [4]. The Sabah state’s prevalence for hypercholesterolemia was 40.9%, hypertension was 26.8% and diabetes mellitus was 14.2% [4]. The prevalence for diabetes was lower in Semporna despite 202 (61.2%) of respondents were found to have abdominal obesity. In comparison, the prevalence of abdominal obesity was 46.4% for the state of Sabah, 48.6% for the country, and 46.2% for rural areas throughout the country [4]. These differences could be attributable to differences in genetics and food intake. It has been demonstrated that indigenous Sarawakians in the northwestern part of Borneo island, had higher adjusted prevalence ratios for metabolic syndrome compared to ethnic Malays and Chinese in Malaysia [25]. The staple food of the Bajau Lauts, a marine nomadic group, was tapioca, rice and fish [26]. The settled Bajau shared similar food culture with the Bajau Lauts. The Bajau Lauts had recently been shown to have genetic variants in the PDE10A gene which resulted in increased spleen sizes [27]. The spleen might have a physiological role in diabetes mellitus as demonstrated by splenectomised patients who had significantly higher mean glucose level (114 mg/dL) than in the control group (90 mg/dL) (p = 0.04) [28]. These findings showed that the coastal communities in Sabah were not similar to Malaysians from other regions and of other ethnic groups in terms of risk factors of NCDs.

It is important to note that the prevalence of hypertension in this rural coastal community was higher than the Sabah state wide prevalence. A similar trend in prevalence of hypertension was noted among adults from coastal areas and islands in China in the 2000s. The estimated prevalence in Chinese coastal areas was 29.1% (95%CI:24.8–33.9) and in island regions was 33.9% (955%CI: 29.4–38.8) compared to China’s pooled, adjusted national prevalence of 20.3% (95%CI:14.1–28.4) [16]. Several studies on hypertension among rural adults in Peninsular Malaysia (also known as West Malaysia) reported comparable prevalence of 26.8–33.6% [2, 3, 29]. The only reported study conducted in three islands within marine park areas in Peninsular Malaysia reported very much lower rates of hypertension (10.7%) and diabetes (0.7%) [30]. In 1985, prevalence of hypertension in rural areas of developing island nations like Vanuatu was lower than that of urban areas. It was 1.1% for rural men and 2.6% for rural women compared to 6.0% for urban men and 4.2% in urban women in Vanuatu [31]. By 2007, 19.7% of households on a rural island in Vanuatu were reporting that they had family members with hypertension or cardiovascular diseases compared to 45.1% in an urban area [32]. Increasing sedentary activities and changes in economic activities seemed to increase prevalence levels in rural islands and rural coastal areas to urban prevalence levels in developing countries such as the South Pacific island countries [32] and China [33].

The occurrence of these three NCDs increased significantly with increasing age, BMI, percent body fat and waist circumference (p < 0.05). Other studies in Malaysia also reported similar observations [2, 3, 29, 34]. As waist circumference increased, the OR for hypertension (OR = 1.09, 95%CI: 1.04–1.14, p < 0.001) and for diabetes (OR = 1.07, 95%CI: 1.00–1.15, p = 0.044) increased (Table 3). In Asian populations, waist circumference might be a more appropriate indicator of obesity and insulin resistance [35]. Obesity had increased in Asian populations with the gap between rural and urban communities narrowing. In a longitudinal study on Filipino women, diabetes was associated with higher waist circumference in relation to higher socioeconomic status and urbanisation [36]. An Indonesian cross-sectional study also showed waist circumference was associated with blood glucose levels [37]. Higher salt intake was associated with higher prevalence of hypertension in coastal rural communities in India [38, 39]. The most recent Malaysian nationally representative data (MANS 2014) on nutrient intake showed that the indigenous population in Sabah had a higher median sodium intake (2026 mg) compared to the national median (1935 mg) [40].

The prevalence of undiagnosed hypertension (24.5%), hypercholesterolemia (40.6%) and diabetes mellitus (5.8%) in this rural coastal community were comparable to the Malaysian national prevalence of undiagnosed NCDs in rural areas, which were 20.7, 40.5 and 9.5% respectively. In contrast, the Malaysian national prevalence of undiagnosed NCDs in urban areas were 16.1, 38.0 and 9.1% respectively [4]. The REDISCOVER study on hypertension in Malaysia found that awareness, treatment and control among hypertensive respondents were significantly lower in rural communities compared to their urban counterparts [5]. Similarly, rural hypertensive adults in China were 49.4% less likely to be detected and 89.5% less likely to be medicated than their urban counterparts [41].

The over-representations of individuals from hardcore poor households in the undiagnosed NCDs (*p* < 0.001) and among those who did not have a health check in the preceding 12 months (*p* < 0.001) are a matter of public health concern. Blood pressure is the easiest to administer compared to blood lipids and blood glucose checks. Yet, only 68.2% of adults in Semporna had access to a blood pressure check in the preceding 12 months. This rate of access had not improved over the 25 years since Gan & Chin reported an access rate of 70.4% for rural populations in Sabah [42]. At that time, the prevalence for hypertension in Kota Belud, another rural community in north-western Sabah was 20.1% [42], which was slightly lower than the prevalence of 24.5% reported for this Semporna study. The high prevalence of undiagnosed NCDs among respondents in the present study could be related to low health consciousness in the community. Results showed that only a small proportion of respondents had undergone health check-up for hypertension (31.8%), hypercholesterolemia (8.2%) and diabetes mellitus (13.0%). NCDs could be asymptomatic which caused individuals to not seek health checks as they perceived themselves as healthy [10]. Low health consciousness in the community could be a result of approaches which were disease focused. When a continuous health programme was designed to be family focused within rural communities in Western Sabah, it was found that many families were unaware of their health problems. Over the period of intervention, those families became more empowered to access health services and community resources such as welfare payments, and there were reductions in blood pressure and blood glucose levels [43].

Semporna has two publicly funded health clinics where such health checks are available for a minimal payment, one on the Semporna mainland and the other on the largest island opposite the mainland. However access to health clinics for NCD detection in this rural coastal community might have been a challenge. There are also eight publicly funded community clinics which are located nearer to the communities that they serve. Unlike the health clinics, community clinics are staffed by community nurses whose focus are on maternal and child health, contraception, immunisation and child developmental assessments [15]. Another issue that required attention is the access to NCD prevention and promotion strategy in rural coastal communities in Sabah. The cost of access to health checks at private clinics is beyond the reach of most individuals in this rural coastal community as median per capita household income was RM83.33/month (≈ USD 20). Public funded NCD prevention and health promotions, whilst they were free, they did not reach those who may not have a need to visit health facilities or did not have access to health services [10]. The NCDP-1 M with its community partnering in NCD risk screening and health promotion were available in the rural coastal community in Semporna, but for various reasons, many adults had not accessed the free NCD risk screening. The high proportion of respondents in the present study who did not have a health check in the past 12 months could be due to a combination of access issues and lack of health awareness.

The situation in Semporna is unique compared to rural coastal communities in other parts of Malaysia and other Southeast Asian countries. Rural coastal communities in Sabah host settled people who might be of similar or different ethnic groups. In the present study, coastal Semporna, the islands off Semporna and its surrounding waters are also home to stateless Bajau Lauts who are of similar ethnicity and to a varying degree, similar culture with the citizen Bajaus. The stateless Bajau Lauts are mostly very poor and are habitually denied access to affordable health care services as Malaysian law does not distinguish the Bajau Lauts as undocumented inhabitants, from refugees and asylum seekers [14].

## Conclusions

Many people in this rural coastal community were unaware that they had high cholesterol and elevated blood pressure. Their lack of health checks could either be attributable to limitations in access to affordable health services for NCD prevention and monitoring, or low health seeking behaviour, or both. Based on the findings, we suggest further studies in (1) the changes in food habits in a rural coastal community and their effects on NCD risks, (2) identifying risk factors for NCDs in rural coastal communities and, (3) the mechanisms for early detection of new cases and provision of adequate treatment. These studies should be conducted in both citizens and undocumented inhabitants who for all matters and purposes are settled within these rural communities.

## Abbreviations

BMI: Body mass index
NCD: Non-communicable disease
NDCP-1 M: NCD Prevention 1 Malaysia
NHMS: National Health and Morbidity Survey
OR: Odd ratio
PLI: Poverty Line Income
RM: Ringgit Malaysia
WHO: World Health Organization
